# Polyploidy Analysis and Attenuation of Oxidative Stress in Hepatic Tissue of STZ-Induced Diabetic Rats Treated with an Aqueous Extract of *Vochysia rufa*

**DOI:** 10.1155/2015/316017

**Published:** 2015-02-11

**Authors:** Izabela Barbosa Moraes, Camilla Manzan-Martins, Neire Moura de Gouveia, Luciana Karen Calábria, Karen Renata Nakamura Hiraki, Alberto da Silva Moraes, Foued Salmen Espindola

**Affiliations:** ^1^Instituto de Genética e Bioquímica, Universidade Federal de Uberlândia, Avenida Pará 1720, 38400-902 Uberlândia, MG, Brazil; ^2^Faculdade São Francisco de Barreiras, BR 135 Km 01 2341, 47800-970 Barreiras, BA, Brazil; ^3^Faculdade de Ciências Integradas do Pontal, Universidade Federal de Uberlândia, Rua 20 1600, 38304-402 Ituiutaba, MG, Brazil; ^4^Instituto de Ciências Biomédicas, Universidade Federal de Uberlândia, Avenida Pará 1720, 38400-902 Uberlândia, MG, Brazil

## Abstract

Diabetes mellitus (DM) is characterized by hyperglycemia and alterations in the metabolism of lipids, carbohydrates, and proteins. Due to its hypoglycemic effect *Vochysia rufa* is frequently used in Uberlandia, Brazil, to treat DM. Despite its popularity, there is little information about its effect on hepatic tissue. Therefore, we evaluated the histoarchitecture, oxidative stress parameters, and polyploidy of liver tissue from streptozotocin- (STZ-) induced diabetic rats treated with aqueous extract of *Vochysia rufa* (AEV). Histology was determined by fixing the livers, processing, and staining with HE. Oxidative stress was determined by evaluating CAT, GPx, and SOD activity in liver homogenates and hepatic mitochondria fraction and by measuring GST, GSH levels and lipid peroxidation (MDA). Polyploidy was determined by subjecting isolated hepatocyte nuclei to flow cytometry. In the diabetic group, GST activity and GSH rates decreased whereas liver homogenate analysis showed that GPx, SOD activity and MDA increased. AEV treatment restored all parameters to normal levels. The oxidative stress analysis of hepatic mitochondria fraction showed similar results. Lower polyploid cell populations were found in the diabetic rat livers, even after glibenclamide treatment. Thus, AEV treatment efficiently reduced hepatic oxidative stress caused by STZ-induced diabetes and produced no morphological changes in the histological analysis.

## 1. Introduction

Diabetes mellitus is a metabolic disorder characterized by hyperglycemia resulting from insufficient secretion of or receptor insensitivity to endogenous insulin [1]. Moreover, DM causes alterations in carbohydrate, protein, and lipid metabolism [2]. Diabetic complications are linked to hyperglycemia-induced oxidative stress which eventually overcomes the endogenous antioxidant defense system through glucose autoxidation, induction of nonenzymatic glycosylation of various macromolecules, and generation of reactive oxygen species (ROS) [3]. The human body possesses several enzymes associated with antioxidant defense and repair mechanisms against oxidative stress, such as catalase (CAT), superoxide dismutase (SOD), glutathione peroxidase (GPx), reduced glutathione (GSH), and glutathione S-Transferase (GST) [4].

The liver is the main detoxifying organ in the body but also plays a central role in metabolic homeostasis [5]. Alterations in hepatic glucose metabolism are associated with diabetes, and changes to many hepatic enzymes occur in diabetic individuals [6].

For years, various people around the world have used medicinal plants to manage diabetes [7–12]. Studies have shown that plants can have beneficial effects on diabetic complications [13, 14], especially on hepatic oxidative stress [14–16].* V. rufa* Mart. popularly known as “sweet bark,” has been used in folk medicine to treat diabetes mellitus type 1 and type 2 in Uberlandia, Brazil. Several species of the genus* Vochysia* have important therapeutic and medicinal properties. Phytochemical characterization of work carried out with the genus led to the isolation of polyphenols and triterpenes [17]. Unlike our study, the main compounds found by Silva [18] present in the methanol extract of* Vochysia* drums were phenolic compounds, coumarins, saponins, and triterpenoids. However, there is not any report about the sugars hitherto; let alone its antidiabetic activity in experimental model of the diabetes. Therefore, the present study investigates the effect preliminary of an aqueous extract of* V. rufa* (AEV) on the hepatic tissue and hepatic mitochondria fraction of diabetic rats by examining GPx, GST, SOD, CAT activity, lipid peroxidation, GSH levels, histoarchitecture, and polyploidy.

## 2. Materials and Methods

### 2.1. Plant Material and the Aqueous Extract

Stem bark of* V. rufa* Mart. was collected from the Cerrado biome in the outskirts of Abadia dos Dourados/MG, Brazil (latitude 18°27′50.5′′ and longitude 47°23′37.2′′), from October 2010 to February 2011. The plant was identified and a voucher specimen deposited (number 58,888) at the* Herbarium Uberlandensis* of the Universidade Federal de Uberlandia. The bark was dried at 40°C and ground to a powder. The aqueous extract was obtained using a common procedure that involves the maceration of 200 g of bark in 1 L of distillated water for 24 h (1 : 5 w/v) at room temperature. The resulting extract was then filtered and centrifuged at 2000 ×g at 4°C, for 15 min. Finally, the supernatant was collected, frozen, and lyophilized.

### 2.2. Quantification of Reducing Sugars

The presence of reducing sugars was determined by the Lane-Eynon method, in which cupric salts in alkaline tartrate solution can be reduced by heating aldoses and ketoses turning into red cuprous salts [19]. In this procedure, 5 mL of solution A and 5 mL of Fehling solution B were transferred to a 250 mL Erlenmeyer flask with the aid of a pipette and, then, 50 mL of distilled water was added for heating until boiling. Then, the test sample was transferred to a 25 mL burette and added dropwise over Fehling's solution, boiling, with continuous stirring until the solution changed from blue to colorless. A reddish residue was formed in the flask bottom and 2-3 drops of methylene blue were added to it, and the titration was completed according to the change of color. The Fehling's solution was previously calibrated with a 0.5% glucose solution (v/v in water), with three replications. Calculation: 100 × *A* × *a*/*P* × *V* = Reducing carbohydrates in % glucose (w/w). The mL of the sample solution = spend the titration; *a* = title Fehling (number of grams of glucose, corresponding to 10 mL of Fehling solution); *P* = *g* of sample (or initial volume of the sample); *V* = volume of the sample spent titration. The simplified formula can be expressed as:

(1) $$\begin{array}{c} \text{Gear carbohydrates into glucose}\left(\%\right)=a\times 100/V. \end{array}$$

A solution of the aqueous extract of* Vochysia* 0.5% was prepared and carried out the same procedures to glucose.

### 2.3. Animals

Adult Wistar male rats (200–250 g) were obtained from ANILAB (Animais de Laboratório Criação e Comércio Ltda-EPP), Paulínia, Brazil. The animals were maintained under standard conditions (22 ± 1°C, 60 ± 5% humidity and a 12 h light : 12 h dark cycle). The animals were fed a commercial pellet diet (65.82% carbohydrate, 5.36% fiber, 21.0% protein, and 4.96% fat) (BioBase, SC, Brazil) and received water* ad libitum*. All procedures for the handling, use, and euthanasia of the animals were approved by the Ethics Committee for Animal Research at the Federal University of Uberlandia, Brazil (CEUA/UFU 060/10).

### 2.4. Induction of Diabetes

The rats were intraperitoneally anesthetized with xylazine/ketamine (1 : 1, v/v). Diabetes was induced in overnight-fasted animals by an intraperitoneal injection (40 mg/kg) of STZ (Sigma-Aldrich, St. Louis, MO. USA) freshly dissolved in 0,01 M citrate buffer (pH 4.5). Citrate buffer was also administered to the animals in the nondiabetic group. The animals were fasted for another 90 min after the injection. Ten days after the STZ injection, a portable glucometer was used to measure fasting blood glucose in blood collected from the tail veins of all animals. Animals with blood glucose levels >250 mg/dL were considered diabetic.

### 2.5. Experimental Procedure

The animals were divided into six groups of 10 animals: a nondiabetic control (ND), a diabetic control (DB), nondiabetics treated with 500 mg/kg of aqueous extract of* V. rufa* (ND-AEV), nondiabetics treated with 6 mg/kg of glibenclamide (ND-GB), diabetics treated with 500 mg/kg of aqueous extract of* V. rufa* (DB-AEV), and diabetics treated with 6 mg/kg of glibenclamide (DB-GB). Body weight was measured at the beginning and end of the study and the animals had free access to food and water. On day 43 of the treatment, the rats were anesthetized with xylazine/ketamine (1 : 1, v/v). Livers were then removed, rinsed in saline solution to remove blood, weighed, and immediately frozen and stored at −80°C for assays (*n* = 6). Livers were also fixed in buffered formaldehyde (10%) for histological analysis (*n* = 4).

### 2.6. Liver Homogenization for Evaluation of Oxidative Stress

Liver samples (1 g) were homogenized on ice in four volumes of homogenization buffer (20 mM Tris-HCl, pH 7.4, 2 mM dithiothreitol, 1 mM benzamidine, 0.5 mM phenylmethanesulfonyl fluoride, 0.5 mM aprotinin, and 0.1 mM pefabloc). Next, the homogenate was centrifuged at 800 ×g for 10 min at 4°C and protein concentrations were then measured by the Bradford method [20].

### 2.7. Mitochondrial Fraction

A 1 g liver sample was homogenized in 4 mL of homogenization buffer (25 mM sucrose, 20 mM HEPES, 1 mM EDTA, pH 7.4, 2 mM dithiothreitol, 1 mM benzamidine, 0.5 mM phenylmethanesulfonyl fluoride, 0.5 mM aprotinin, and 0.1 mM Pefabloc). The homogenate was then filtered and centrifuged at 800 ×g at 4°C for 10 min. The supernatant was centrifuged again at 10,000 ×g at 4°C for 10 min to obtain the mitochondrial fraction. Finally, the mitochondrial pellet was suspended by the same buffer and stored at −80°C for analysis.

### 2.8. Oxidative Stress Analysis

Lipid peroxidation was biochemically assessed by determining malondialdehyde levels (MDA) [21]. Catalase (CAT) activity was measured according to the method of Aebi [22]. Superoxide dismutase (SOD) was measured as described by Boveris [23]. Glutathione peroxidase (GPx) activity was assayed by the method described by Flohe and Gunzler [24], and glutathione S-transferase (GST) activity was measured according to Habig et al. [25]. Reduced glutathione (GSH) was measured with 2-nitrobenzoic acid (DTNB) reagent as described by Beutler et al. [26]. Total protein in the tissue homogenate and mitochondrial fraction was estimated by the method of Bradford [20].

### 2.9. Histological Analysis

Histology was determined by fixing the livers in 10% phosphate-buffered formalin, processing, embedding in paraffin, and then staining with H&E. Hepatic polyploidy was assessed cytophotometrically by subjecting liver tissue to a Feulgen reaction with room-temperature hydrolysis (4 N HCl). Stained slides were captured on a LEICA DM500 microscope, equipped with a LEICA Application Suite (Version 1.8.1) camera. Digitalized images were analyzed using Image J open source software and the integrated optical density parameter, which shows the average DNA quantity in the nuclei of each population.

### 2.10. Flow Cytometry

Hepatocyte nuclei were isolated according to the method described by Blobel and Potter [27]. Immediately after tissue homogenization, using a Potter homogenizer in 0.25 M sucrose in TKM (Tris 0.02 M, KCl 0.025 M, and MgCl_2_ 0.005 M) buffer, the homogenate was filtered, mixed with 2.3 M sucrose in TKM, and applied to 2.3 M sucrose in TKM solution. This process produced a gradient that was centrifuged at 124,000 ×g at 4°C for 30 min. Pelleted nuclei were suspended in TKM buffer and stained with propidium iodide for analysis by flow cytometry (BD Accuri C6 Flow Cytometer). Analysis was then performed using FlowJo software.

### 2.11. Statistical Analysis

Oxidative stress data were analyzed by the Student *t*-test (SigmaStat 3.5 software, Systat Software Inc., IL, USA) and expressed as means ± S.E.M. Medians of the flow cytometry data were analyzed in Minitab software using Mood's Median Test. A *P* value < 0.05 was considered significant for all tests.

## 3. Results

### 3.1. Quantification of Reducing Sugars

The presence of reducing sugars was determined by the Lane-Eynon method, wherein the* Vochysia* extract contained 70% reducing carbohydrates. Furthermore, we observed that there is no absorption spectrum in HPLC, as analyzed in HPLC profile, where there is no separation of the compounds; however, these molecules have not yet been isolated and characterized.

### 3.2. Effects of AEV on Body Weight, Blood Glucose Level, and Liver Weight in STZ-Induced Diabetic Rats

After 43 days of AEV treatment, blood glucose levels of the diabetic group decreased (314.6 ± 87.3 mg/dL), although it is not significantly compared to the diabetic control group (535 ± 43 mg/dL). However, glucose levels of glibenclamide treated diabetics were significantly lower than diabetic controls (97 ± 19 mg/dL).

The mean body weight of diabetic rats was significantly lower than that of nondiabetic controls (Table 1). Neither AEV nor glibenclamide treatment changed this parameter in the nondiabetic and diabetic groups. Liver weight decreased after diabetes but tended to revert to levels observed in nondiabetic animals after AEV treatment and reverted completely after glibenclamide treatment. Liver weight was higher in diabetic animals. These results indicate that diabetes decreased body mass more drastically than liver mass and that while AEV and glibenclamide were unable to restore body weight to normal levels, these treatments were able to increase liver weight back to control values.

### 3.3. Oxidative Stress Analysis

MDA, SOD, and GPx activity increased and GST and GSH activity decreased after diabetes (Table 2). AEV treatment reverted all these changes except SOD. In diabetic rats, glibenclamide treatment restored GPx and SOD to normal and tended to restore GSH, GST, and MDA. In some cases (CAT, GPx, and SOD), AEV treatment produced opposite results in nondiabetic and diabetic rats.

CAT and SOD activities were significantly lower in the hepatic mitochondrial tissue (Table 3) of the diabetic control group than in the nondiabetic control. Both CAT and SOD activity increased in the diabetic group treated with AEV, but only CAT activity increased in the diabetic group treated with glibenclamide. Finally, GPx activity was higher in the liver mitochondria of the diabetic group, but lower in both treated diabetic groups.

### 3.4. Histological Analysis

**Figure 1:**
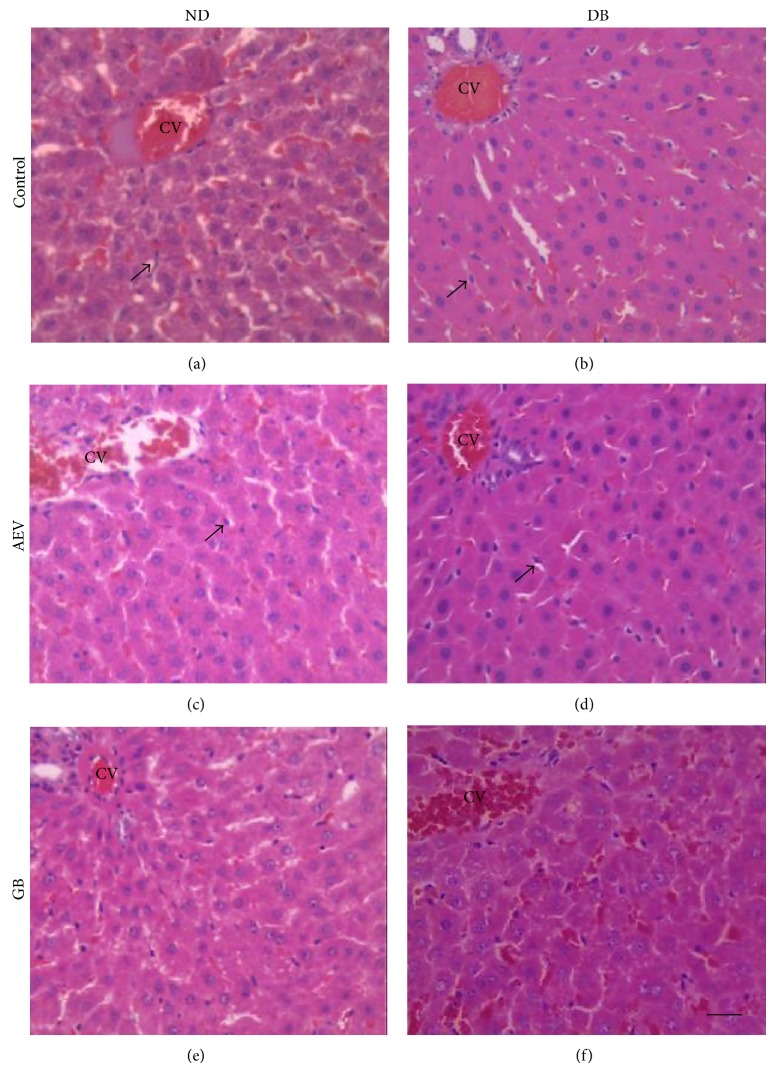
Photomicrographs of liver sections from nondiabetic control rats (ND) (a), diabetic control (DB) (b), nondiabetic treated with aqueous extract of* V. rufa* (AEV) (c), diabetic treated with AEV (d), nondiabetic treated with glibenclamide (GB) (e), and diabetic treated with (GB) (f). H&E. (Bar = 25 *μ*m.) CV: central vein; Kupffer cells with a normal aspect are indicated by arrows.

Analysis of HE-stained liver slices (Figure 1) showed preservation of liver histoarchitecture, hepatocytes with a normal cytoplasmic eosinophilic aspect, and one or two nuclei with loose chromatin and evident nucleolus. No evidence of inflammation, necrosis, fibrosis, proliferation, destruction, or parenchyma regeneration and therefore no visual signs of hepatotoxicity were observed.

### 3.5. Polyploidy

Hepatic polyploidy was estimated by analyzing isolated hepatocyte nuclei via flow cytometry. The results showed that streptozotocin induced diabetes reduced the ratio of tetraploid nuclei to diploid nuclei (Table 4). In other words, diabetes reduced polyploidy relative to the nondiabetic control group. Polyploidy was not statistically higher in the diabetic group treated with EAV than in the control; however, the data did show a tendency for polyploidy to increase towards normal rates after this treatment. On the other hand, glibenclamide treatment of both diabetic and nondiabetic rats significantly decreased numbers of tetraploid nuclei relative to diploid nuclei. Images of Feulgen-stained liver slices confirmed these results (data not shown).

## 4. Discussion

STZ-induced diabetes is one of the most commonly used experimental models of human diabetes used in research [28]. STZ induces hyperglycemia by destroying pancreatic *β*-cells and consequently reducing insulin production. Hyperglycemia is associated with metabolic change in the liver, especially loss of body weight and antioxidant imbalance [29, 30], which occurred in the present study. Medicinal plants can reduce the effects of diabetes, mainly because of their hypoglycemic properties and capacity to counteract oxidative stress [15, 16, 31].

Despite its popularity, information is lacking on the efficiency of* V. rufa* as a treatment for diabetes. Nevertheless, several species of the* Vochysia* genus have important therapeutic and medicinal properties. Phytochemical characterization of species of this genus has isolated polyphenols and triterpenes [17]. However this study demonstrated the presence of sugars in the aqueous extract of* V. rufa*. Similar to our results, [32] demonstrated that* Coptis chinensis* contains 96.3% carbohydrate, 4.8% uronic acid, and 0.61% protein and is mainly composed of glucose, arabinose, xylose, galactose, and galacturonic acid. In recent years, the antidiabetic effect of polysaccharide has widely been reported [33–35]. Zhang et al. [36] demonstrated that the orally administered* T. cuspidata* polysaccharides had an effective hypoglycemic effect and could effectively alleviate the impaired oxidative stress in the kidney and liver of the diabetic rats induced by STZ. Jiang et al. [32] demonstrated that* Coptis chinensis* polysaccharides could produce a potent and efficacious antidiabetic effect and antioxidant activity in diabetic mice.

Increased lipid peroxidation, as observed in the current study, is associated with increased oxidative stress, especially in the STZ-induced diabetic model [31, 37, 38]. Under hyperglycemic conditions, glucose autooxidizes and produces superoxide, leading to the production of free radicals that in turn cause lipid peroxidation in lipoproteins [15]. In the present study MDA formed in the diabetic group. In contrast, MDA formation was lower in the diabetic group treated with AEV. Similar results were found for other researchers [14]. CAT is produced by peroxisomes and plays a role in cell defense by breaking down hydrogen peroxide (H_2_O_2_) into H_2_O and O_2_. No differences in liver homogenate analysis were observed; however, CAT activity was lower in the mitochondria fraction of the diabetic control group. Low MDA content combined with significant increases in catalase activity was observed in diabetic rats treated with the extract, confirming the antioxidant effect of this treatment. The extract may therefore lead to significant elimination of free radicals caused by diabetes.

The SOD oxide-reductase group is responsible for superoxide anion (O_2_^−^) dismutation into molecular oxygen and water. In the present study, SOD activity was higher in the liver homogenates of STZ-induced diabetic rats in response to greater hepatic oxidative stress [16, 29]. However, SOD activity was lower in the hepatic mitochondria fraction of the diabetic group. Kapoor and Kakkar [39] showed that, in cultured hepatocytes, MnSOD (mitochondrial SOD) and CuSOD (cytosolic SOD) are differentially expressed when exposed to high glucose concentrations (40 mM). Although high-glucose induces low MnSOD expression, it also leads to high CuSOD expression. These differences could be explained by potential differences in SOD concentration between liver homogenates and hepatic mitochondria. Treatment with* V. rufa* extract increases SOD activity in liver mitochondria fraction, which may protect against oxidative injury.

GPx activity has been shown to be higher in liver homogenates than in hepatic mitochondria fraction [12, 40], whereas GSH levels have been shown to be lower [15, 38]. The glutathione system is the main intracellular antioxidant mechanism. It is dependent on GSH concentration and the GSH/GSSG ratio, which is regulated by de novo synthesis of glutathione in a redox cycle mediated by reactions controlled by GPx and GR. Additionally, lower GSH levels have been considered as the hallmark of oxidative stress [12]. Furthermore, high GPx activity in diabetic animals is related to higher oxidative stress in the liver. The decreases in GST activity for the diabetic group in the present study corroborate the findings of other studies [38]. GST is a family of isoenzymes that participate in conjugating toxic electrophiles to GSH. Therefore, decreased GST activity in diabetic rats may be related to lower GSH availability.

Abundant clinical evidence demonstrated that the diabetes correlated closely with oxidative stress, resulting in an increased ROS production or a reduction in the antioxidant defense system [41]. Oxidative stress decreased after AEV treatment as evidenced by the reduction of lipid peroxidation and GPx activity to normal levels. GST activity increased, protecting cells against the toxic effects of harmful compounds and preventing oxidative damage [42]. Higher SOD and CAT activity in the mitochondria fraction of diabetic treated rats is related to nondiabetic control levels and high GSH levels. Many reports on medicinal plant treatments for diabetes show reduced oxidative stress in the liver [15, 28, 43]. These results indicated an obvious antioxidant effect of* V. rufa* on STZ-induced diabetic rats in hepatic tissue due to presence of polysaccharides. Polysaccharide mechanism involved in antidiabetic activity may include increased levels of serum insulin, reduction of blood glucose level, and improved tolerance of glucose [44]. This is done by our group a study to evaluate the effect of* V. rufa* extract in pancreas induced diabetic rats. In this study we evaluated the ability of the extract to increase insulin secretion.

Polyploid cells form is present in several tissue types [45], including liver tissue. Gentric et al. [46] described three principal effects of polyploidy on liver tissue. First, polyploidy could protect hepatocytes from genotoxic damage by increasing the number of copies of functional genes. This function may be especially important for an organ whose primary role is to metabolize and eliminate toxic compounds. Second, polyploidy could be an economical solution to growth problems that occur when an organ works within its capabilities, avoiding the great energy demands of cell division. Finally, polyploidy could alter the expression of specific genes. Our study showed reduced levels of polyploidy in STZ-induced diabetic rats. If this phenomenon benefits the tissue, diabetes could leave the hepatic tissue unprotected. Celton-Morizur et al. [45] showed that tetraploid hepatocyte numbers decreased in rats treated with streptozotocin. This study also demonstrated that insulin regulates the genesis of binucleated tetraploid liver cells. Because STZ destroys beta-cells, insulin production decreases to the point that liver development is impaired.

Increased polyploidy in the liver has been associated with several health conditions, including aging. Ghiraldini et al. [47] demonstrated that NOD mice with severe hyperglycemia have greater ploidy levels than nondiabetic mice. Our contradictory results might suggest that the ploidy parameter is determined purely by STZ administration, and not by hyperglycemia. Therefore, the toxic effects of STZ may directly affect hepatic metabolism and thus impair the capacity of hepatocytes to increase their degree of ploidy via endoreplication.

STZ is a glucosamine-nitrosourea compound which enters cells through the GLUT2 transporter and interacts with DNA causing severe mutations. Thus, STZ can be used to kill cancerous beta-pancreatic cells when surgery is inviable. However, GLUT2 is also the primary glucose transporter in liver cells, allowing it to enter hepatocytes [48]. Approximately 15–67% of patients treated with streptozotocin chemotherapy develop some kind of hepatocellular damage [49]. Although no morphological evidence of hepatotoxicity was observed in the present study, STZ treatment may still have directly altered the liver parameters investigated. However, given that AEV treatment reverted many alterations, it indicates that STZ-induced hyperglycemia may also have influenced hepatic change.

## 5. Conclusion

We showed that STZ-induced diabetes increased oxidative stress, led to higher relative liver weight, and reduced liver polyploidy, suggesting that these changes may have been caused by the pathology of diabetes in the liver. Nevertheless, STZ could also be directly responsible for these alterations given that the drug itself is hepatotoxic. The action of the sugars presents in AEV extract improved the antioxidant enzymes activity and the elevated lipid peroxidation in STZ-induced diabetic rats implied its free radical scavenging potential and hence it has the ability to prevent diabetic associated complication. However, since AEV treatment reduced the deleterious effects on liver tissue, it seems that both STZ (principally) and diabetes (secondarily) are responsible.

## Figures and Tables

**Table 1:** Effects of aqueous extract of *Vochysia rufa* and glibenclamide on body and liver weight in STZ-induced diabetic and nondiabetic rats.

	CAT *μ*g/seg	GPx mmol/min/mL	GSH mM	GST *μ*mol·min^−1^·g^−1^	SOD U/mg protein	MDA nmol/mg protein
ND	334.5 ± 23.1	2.2 ± 0.2	3.7 ± 0.3	121.2 ± 6.9	0.6 ± 0.1	1.5 ± 0.1
ND-AEV	439.6 ± 37.8^∗a^	4.3 ± 0.5^∗a^	4.8 ± 0.3^∗a^	111.2 ± 4.5	2.8 ± 0.7^∗a^	1.4 ± 0.1
ND-GB	388.9 ± 28.7	2.4 ± 0.2	2.6 ± 0.3^∗a^	105.3 ± 0.8	0.6 ± 0.1	1.5 ± 0.2
DB	348.2 ± 23.3	3.8 ± 0.3^∗a^	2.7 ± 0.3^∗a^	84.8 ± 5.1^#a^	1.3 ± 0.2^∗a^	1.9 ± 0.1^∗a^
DB-AEV	312.1 ± 22.6	2.3 ± 0.3^∗b^	3.7 ± 0.2^∗b^	121.7 ± 15.7^∗b^	1.2 ± 0.1	1.3 ± 0.1^∗b^
DB-GB	320.9 ± 20.0	2.4 ± 0.2^∗b^	3.2 ± 0.2	93.6 ± 7.4	0.6 ± 0.1^#b^	1.6 ± 0.2

Data are shown as means ± S.E.M.; *n* = 6; ^*^ *P* < 0.05; ^#^ *P* < 0.005.

^a^Compared with a nondiabetic control group.

^b^Diabetic treated group compared with a diabetic control.

ND: nondiabetic. DB: STZ-induced diabetic. AEV: treated with aqueous extract of *Vochysia rufa*. GB: treated with glibenclamide.

**Table 2:** Measurements of oxidative stress parameters in liver homogenates of STZ-induced diabetic and nondiabetic rats, treated or not with AEV and glibenclamide.

	Initial BW (g)	Final BW (g)	LW (g)	Relative LW (%)
ND	264.6 ± 0.8	356.9 ± 10.5	11.7 ± 0.5	3.3 ± 0.1
ND-AEV	268.7 ± 3.85	350.5 ± 9.4	9.9 ± 0.3^#a^	2.9 ± 0.1^#a^
ND-GB	269.3 ± 3.8	355.5 ± 8.9	10.1 ± 0.3^#a^	2.8 ± 0.1^#a^
DB	237.0 ± 8.1	225.9 ± 8.4^∗a^	9.0 ± 0.2^∗a^	4.0 ± 0.1^∗a^
DB-AEV	224.8 ± 10.9	220.2 ± 10.0	9.6 ± 0.2	4.3 ± 0.2
DB-GB	231.7 ± 9.9	249.0 ± 14.6	12.3 ± 0.7^∗b^	4.9 ± 0.4

Data are shown as means ± S.E.M.; *n* = 10; ^*^ *P* < 0.001; ^#^ *P* < 0.05.

^a^Compared with a nondiabetic control group.

^b^Diabetic treated group compared with a diabetic control.

ND: nondiabetic. DB:STZ-induced diabetic. AEV: treated with aqueous extract of *Vochysia rufa*. GB: treated with glibenclamide. BW: body weight. LW: liver weight.

**Table 3:** Evaluation of CAT, GPx, and SOD activity in the hepatic mitochondria of STZ-induced diabetic and nondiabetic rats treated with aqueous extract of *Vochysia rufa* and glibenclamide.

	CAT *µ*g/seg	GPx mmol/min/mL	SOD U/mgprotein
ND	484.2 ± 41.2	2.4 ± 0.3	1.7 ± 0.17
ND-AEV	759.3 ± 88.7^∗a^	5.66 ± 0.4^*€*a^	1.8 ± 0.32
ND-GB	689.3 ± 80.7^∗a^	3.6 ± 0.5	1.1 ± 0.08^∗a^
DB	352.9 ± 14.1^∗a^	5.1 ± 0.3^#a^	0.8 ± 0.15^∗a^
DB-AEV	465.4 ± 24.1^∗b^	3.9 ± 0.3^*€*b^	1.28 ± 0.08^#b^
DB-GB	486.3 ± 31.1^∗b^	1.6 ± 0.1^*€*b^	0.9 ± 0.09

Data are shown as means ± S.E.M.; *n* = 6; ^*^ *P* < 0.05;  ^#^ *P* < 0.005; ^*€*^ *P* < 0.001.

^a^Compared with a nondiabetic control group.

^b^Diabetic treated group compared with diabetic control.

ND: nondiabetic. DB: STZ-induced diabetic. AEV: treated with aqueous extract of *Vochysia rufa*. GB: treated with glibenclamide.

**Table 4:** Streptozotocin-induced diabetes mellitus alters ploidy levels in rats.

Group	ND	ND-AEV	ND-GB	DB	DB-AEV	DB-GB
2n/4n	0.63^a^	0.53^a,c^	0.79^b,c^	0.86^b,c^	0.74^a,b,c^	1.81^d^

2n/4n, medians of the ratio between diploid and tetraploid cells; *n* = 6; different letters in the same line indicate statistical significance (*P* < 0.05).

ND: nondiabetic. DB: STZ-induced diabetic. AEV: treated with aqueous extract of *Vochysia rufa*. GB: treated with glibenclamide.
